# Classical Batch Distillation of Anaerobic Digestate to Isolate Ammonium Bicarbonate: Membrane Not Necessary!

**DOI:** 10.3390/bioengineering11111152

**Published:** 2024-11-15

**Authors:** Alejandro Moure Abelenda, Jonas Baltrusaitis

**Affiliations:** 1 School of Engineering, Lancaster University, Lancaster LA1 4YW, UK; 2 Soil Quality Assessment Research Group, Department of Soil Science and Agricultural Chemistry, Universidade de Santiago de Compostela, Avenida de Vigo, s/n, 15782 Santiago de Compostela, Spain; 3 Department of Chemical and Biomolecular Engineering, Lehigh University, B336 Iacocca Hall, 111 Research Drive, Bethlehem, PA 18015, USA; job314@lehigh.edu

**Keywords:** crystallization, commercial-grade fertilizer, manure management, circular economy, mitigation technology

## Abstract

The excessive mineralization of organic molecules during anaerobic fermentation increases the availability of nitrogen and carbon. For this reason, the development of downstream processing technologies is required to better manage ammonia and carbon dioxide emissions during the storage and land application of the resulting soil organic amendment. The present work investigated classical distillation as a technology for valorizing ammoniacal nitrogen (NH_4_^+^-N) in anaerobic digestate. The results implied that the direct isolation of ammonium bicarbonate (NH_4_HCO_3_) was possible when applying the reactive distillation to the food waste digestate (FWD) with a high content of NH_4_^+^-N, while the addition of antifoam to the agrowaste digestate (AWD) was necessary to be able to produce an aqueous solution of NH_4_HCO_3_ as the distillate. The reason was that the extraction of NH_4_HCO_3_ from the AWD required a higher temperature (>95 °C) and duration (i.e., steady state in batch operation) than the recovery of the inorganic fertilizer from the FWD. The titration method, when applied to the depleted digestate, offered the quickest way of monitoring the reactive distillation because the buffer capacity of the distillate was much higher. The isolation of NH_4_HCO_3_ from the FWD was attained in a transient mode at a temperature below 90 °C (i.e., while heating up to reach the desired distillation temperature or cooling down once the batch distillation was finished). For the operating conditions to be regarded as techno-economically feasible, they should be attained in the anaerobic digestion plant by integrating the heat harvested from the engines, which convert the biogas into electricity.

## 1. Introduction

The planetary boundaries framework has identified nine processes that should be at a stable level to make possible life on Earth. Thereby, any disturbance of these processes by human activity is closely monitored in the current Anthropocene epoch [1]. Particularly, the biosphere (which is made up of the parts of Earth where life exists [2,3]) constitutes a major factor in regulating the climate change derived from the carbon dioxide (CO_2_) concentration in the atmosphere and the biogeochemical flow of nitrogen (N) [1,4]. The anthropogenic biological N fixation currently limits the introduction of new reactive N to the Earth system, and for this reason, the regional distribution of N fertilizer and reuse of nitrogenous materials [5] are critical to maintain a stable planetary boundary [1] and enhance the circular economy [6]. Anaerobic digestion (AD) is a well-known biodegradable organic waste treatment that produces the energy-rich stream of biogas (~65 vol.% CH_4_ and ~35 vol.% CO_2_ [7]), which is typically fed to combined heat and power (CHP) engines for the production of electricity, and a valuable soil organic amendment with a high content of mineralized nutrients [8]. During the anaerobic fermentation, the organic N is converted to ammoniacal nitrogen (NH_4_^+^-N), due to the microbial activity. Although NH_4_^+^-N is more readily available to plants, it is also more prone to be lost by volatilization [9]. Although the best approach would be to recover the excess of NH_4_^+^-N as a chemical grade compound, simply removing the excess of N by depleting the anaerobic digestate with electrocoagulation to make it a more stable fertilizer is less economically beneficial, but still necessary to minimize the contamination of the environment [10].

Distillation is among the most profitable technologies for recovering ammonia (NH_3_) from aqueous solutions (e.g., anaerobic digestate, wastewater, etc.) [11,12]. Interestingly, the classical distillation method without membranes is not widely regarded despite the vapor pressure of NH_3_ (614.4 kPa at 10 °C [13,14]), which is much higher than that of water (1.2 kPa at 10 °C [13,14]). The system NH_3_-H_2_O is not azeotropic and the use of membranes is not compulsory to overcome the problem of identical boiling points [15], although complete separation is not feasible at atmospheric pressure with the conventional distillation method [16,17,18]. The most investigated processes are those which involve stripping NH_3_ off the H_2_O vapor by chemical (e.g., sulfuric acid trap) or mechanical (e.g., gas-permeable membrane [19,20]) methods. Lü et al. [20] highlighted that the membrane-based technologies are as relevant as stripping or ion exchange processes for NH_3_ recovery from the anaerobic digestate. According to Lü et al. [20], the combination of bioelectrochemical processes and membrane contactor methods avoids the pH adjustment, which is an advantage compared to the stripping that requires the addition of alkali and increases the cost of the technology for recovering NH_3_ [11,12,21]. The current trend denotes the membrane technology as a distillation process [22,23,24,25,26,27,28], although most of these processes do not imply the formation of a condensate [27]. The distillate is regarded as the liquid that flows on the other side of the membrane [23], with this aqueous solution not necessarily being the permeate, and even using a sulfuric acid (H_2_SO_4_) aqueous solution to better retain NH_3_ [26]. In the membrane contactors, there is an association of a vapor–liquid phase change and fugacity equilibrium, and permeates are often generated by a chemical ion exchange/solubility with the membrane surface constituents or by pore size filtration. Karanasiou et al. [29] combined membrane technology and cooling to produce a N-rich distillate. These processes require the use of a vacuum to enable a mass transfer throughout the membrane, which increases the cost of the process [25,29]. According to Aiouache and Goto [15], the incorporation of a membrane in a distillation column leads to complex interactions between the vapor–liquid equilibria, chemical reaction kinetics, energy, and mass transfer. Particularly, Aiouache and Goto [15] aimed to hybridize a pervaporative membrane and a reactive distillation column.

In general, all these “distillation” processes are expected to have high capital and operational expenditure (CAPEX and OPEX) costs. Problems of fouling formation have been reported when the digestate is in direct contact with the membrane [27,28]. In the process of Charfi et al. [27], the anaerobic digestate could reach the membrane by excessive foam formation and, in the processes of Aquino et al. [28] and Jacob et al. [22], with direct contact membranes, the dirtiness problems were already anticipated. The key parameters of this novel “distillation” membrane are different from those of the conventional batch distillation, and this could be seen as unnecessarily dispensing on reputed techniques that have been developing for more than a century: for example, the tuning heating rate, boil-up rate, and reflux ratio to improve the separation efficiency of a non-ideal mixture. It is important to highlight that the direct contact membranes can recover a variety of nutrients, in addition to NH_4_^+^-N, for example, phosphorus, sulfur, and potassium [24]. Gas-permeable membranes could be more suitable for very diluted waste streams because they allow the depletion of NH_4_^+^-N [24]. However, in the case of organic manures widely used for the fertilization of field crops, the membrane technology might not be the most suitable application to stabilize and to reduce the content and availability of nutrients. This slight change in the composition of the soil organic amendment material improves its slow-release properties demanded by the agroindustry’s policies to minimize the environmental pollution. The enhancement of the circular economy with this strategy would be given by the additional income stream of the recovered nutrients and by the resulting manure demanded by farmers more suitable for fertilization purposes [9,30,31,32,33,34,35,36,37,38,39].

Wang et al. [40] described the subsequent scrubbing of the gaseous stream of NH_3_ and CO_2_ stripped from the anaerobic digestate at a slightly higher pressure. The stripped gas, which contained CO_2_, CH_4_, and NH_3_, was introduced into the ammonia absorption reactor with the solution of H_2_SO_4_. This was in line with the Burke’s process [41,42,43], where low pressures (0.25–0.75 bar) were more suitable for NH_3_ and CO_2_ stripping off the anaerobic digestate. Burke [41,42,43] used a gas deficient in CO_2_ and NH_3_ such that the partial pressure of NH_3_ and CO_2_ in the stripping gas was less than the partial pressure of the stripping gas. Wang et al. [40] investigated the effect of the CO_2_ content (40%, 20%, and 10%) and temperature (70, 80, and 90 °C) on NH_3_ removal from a biogas slurry. There is no clear specification for the pressure to promote the formation of solid ammonium bicarbonate (NH_4_HCO_3_), besides that the temperature should be lower than 36 °C and the gaseous stream should have the appropriate ratio of NH_3_ to CO_2_. Temperatures of 12 °C and below 30 °C were specified by Drapanauskaite et al. [16] and Wang et al. [40], respectively, for the crystallization of NH_4_HCO_3_. Both Burke [39] and Drapanauskaite et al. [16] emphasized the importance of maintaining a temperature differential between the stripping chamber/distillation column and the precipitation chamber/crystallizer. Wang et al. [40] investigated the effect of the temperature (5, 10, and 15 °C), gas flow rate (0.25, 0.5, and 0.75 L/min), and agitation speed (0, 300, and 600 rpm) on the formation of crystalline ammonium in the absorption step, including NH_4_HCO_3_, ammonium carbonate ((NH_4_)_2_CO_3_), and ammonium carbamate (NH_2_COONH_4_). Burke [41] suggested to increase the ratio of NH_3_ to CO_2_ by not treating all the biogas produced during the AD (to reduce the quantity of CO_2_ delivered to the precipitation chamber) or using a feedstock with a greater N content for the AD. In the process of Wang et al. [40], the ammonium bicarbonate was used for the treatment of the feedstock (i.e., crop residues); thus, a significant portion of NH_3_, which was not removed from the downstream valorization process as (NH_4_)_2_SO_4_, was used as a catalyst to enable the combined process of AD with downstream valorization, similar to the role of NH_3_ in the Solvay process [44,45,46]. NH_4_HCO_3_ was heated above 50 °C to promote its decomposition and the formation of CO_2_; thus, the main components of the solution would be NH_4_OH and (NH_4_)_2_CO_3_ [40]. Wang et al. [40] would need to remove some NH_4_HCO_3_ from the recirculation loop; otherwise, NH_3_ would build up in the system when the process deals with feedstock with a high content of organic N. The approach of the combined production of NH_4_HCO_3_ and (NH_4_)_2_SO_4_ is opposite to the comparison made by Drapanauskaite et al. [16], who considered the production of these two materials (i.e., NH_4_HCO_3_ and (NH_4_)_2_SO_4_) as completely different routes for the valorization of an anaerobic digestate. Also, Ukwuani and Tao [47] saw NH_4_HCO_3_ as a contaminant of the (NH_4_)_2_SO_4_, when the H_2_SO_4_ in the aqueous solution was largely consumed and the pH increased above 7. It is necessary to clarify that although Ukwuani and Tao [47] mentioned the formation of (NH_4_)_2_CO_3_, as Möller and Müller [48] clarified, the formation of this solid compound is unlikely [49].

The use of the carbonic acid already contained in the anaerobic digestate as an endogenous scrubbing agent seems one of the most promising technologies for managing NH_4_^+^-N [49,50]: NH_3_ fixation, the production of solid NH_4_HCO_3_ inorganic fertilizer, and a commercial product [51]. The removal of NH_4_HCO_3_ via classic distillation is important for the further stabilization of the anaerobic digestate (liquor) [52] and for process intensification: coupling the pasteurization and distillation operations by making good use of any heat generated in the AD plant, for example, harvesting the heat of the engines where the biogas is burned to produce electricity. Additionally, it was considered that the traditional distillation method has not been exploited correctly for this particular application of improving the handling of organic manures. The present work exposes the potential of the classic batch distillation to treat different types of anaerobic digestate and to isolate NH_4_HCO_3_ crystals. This investigation focused on the processing conditions to be as economical as possible by aiming for a minimum cost of the thermal separation and a need for downstream operations to recover the NH_4_HCO_3_ crystals. Furthermore, the stability and availability of the valorized inorganic fertilizer to crops and to prevent pollution swapping was also described, based on empirical evidence.

## 2. Materials and Methods

### 2.1. Samples of Anaerobic Digestates

**Figure 1:**
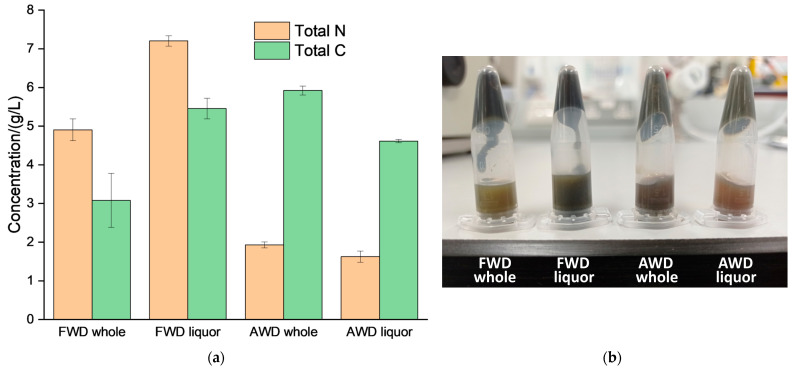
(**a**) Characterization of total N and total C of the different types of anaerobic digestates. (**b**) Liquid fractions isolated by settling at 14,000 rpm for 1 min, and these were diluted 100 times with distilled water before being fed to the TOC-L Shimadzu^®^.

Different types of anaerobic digestates were considered for the present investigation: a food waste digestate (FWD) and an agrowaste digestate (AWD). As reported in previous investigations, the FWD had more than double NH_4_^+^-N than the AWD [32,35,36] due to the greater content of amino acids in the feedstock [8] (Figure 1). Due to the greater content of NH_4_HCO_3_ in the FWD (Figure 1), it was possible to isolate this solid compound directly (this implied the production of a distillate of 100% purity). Figure 1 shows the result of centrifuging the samples of whole digestates and liquors at 14,000 rpm for 1 min to create homogenous supernatants that can be diluted and characterized with the TOC-L Shimadzu^®^ (Shimadzu Europa GmbH, Duisburg, Germany), to determine the contents of total N and total carbon (C) via combustion of the samples and detection of CO_2_ and NO gases. The quantification of the concentration of N and C in the supernatants agreed with the visual inspection (Figure 1). The liquor of FWD had greater concentrations of N and C than the whole FWD, which had not been previously subjected to the solid–liquid separation. This implies that the type of N and C in the FWD mainly prefers the water-soluble fraction, and for this reason, the supernatant of the FWD liquor looks darker than that of the whole FWD (Figure 1). On the other hand, the opposite was found to be true for the AWD, where the raw digestate had a darker supernatant than that of its liquor (Figure 1). The whole AWD was thicker than the whole FWD because the fiber made more stable colloids, which were suspended in the aqueous solution.

### 2.2. Conventional Batch Distillation Setup

**Figure 2:**
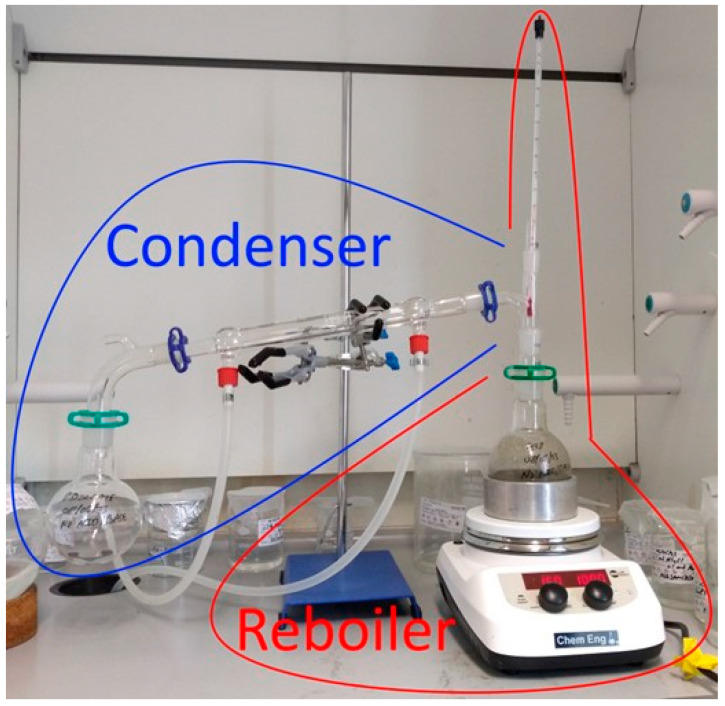
Classical batch distillation setup employed to investigate the isolation of NH_4_HCO_3_ out of anaerobic digestate via reactive distillation, involving desorption, absorption, and deposition.

The processing of the AWD and FWD (whole digestates, comprised of the solid and liquid fractions, and the liquid fractions alone (i.e., liquors)) via batch reactive distillation [15,53] were evaluated with the apparatus described elsewhere [54]. For this reason, different configurations of the batch distillation setup were assessed to gather the crystals of NH_4_HCO_3_ in a more convenient manner. The classical batch distillation apparatus [54] can be divided into reboiler and condenser (Figure 2), which would be comparable to at least 2 plates of a distillation column. Additionally, the point where the temperature of the vapors was measured represented another plate, as continuous dropping of condensate around the thermometer’s bulb could be seen during the operation [15,55]. The reboiler consisted of a ceramic heating plate with magnetic stirring function, an aluminum block, and a round-bottom (RB) flask containing a magnetic stirrer in addition to the anaerobic digestate. Continuous stirring was maintained at a low rate throughout the distillation to ensure the homogenous heating of the anaerobic digestate. The aluminum heating block surrounding the content of the RB flask had the same function of controlling the heat transfer. For each batch assay, approximately 80 g of anaerobic digestate were fed in the 250 mL RB flask, as this ensured that the level of fullness was the same as the height covered by the aluminum heating block. Therefore, there was no overheating zone, while sufficient headspace was available to allow any foam formation and equilibria between the different phases. Different types of antifoam agents provided by Blackburn Chemicals Ltd. (Lancashire, UK) were tested: Dispelair^®^ DP 681 3281 L and Dispelair^®^ K989 4308 L. Dispelair^®^ DP 681 (mixture of esters of alkoxane copolymers [56]) was a general-purpose foam control agent for use in a wide range of aqueous systems. It is particularly effective in effluent plants which employ AD. According to Balckburn Chemicals Ltd. [57], levels of addition range typically between 1 and 10 ppm and should be optimized empirically for each aqueous solution by trial and error. Dispelair^®^ K989 (emulsion of polydimethyl siloxane [58]) was a general-purpose food-grade foam control agent for use in a wide range of aqueous systems. It was particularly suitable for pharmaceutical processes, fermentation, food-grade plastics recycling, and food processing applications such as vegetable processing and starch manufacture. According to Blackburn Chemicals Ltd. [59], levels of addition are typically between 10 and 100 ppm and should be optimized by trial and error. After ensuring proper mixing of the digestate and the antifoam [60], the minimum stirring (50 rpm) was maintained as alternative to anti-bumping agents, which would foster nucleation sites and the formation of small bubbles, but chemically affect the composition of the anaerobic digestate and increase the operating expenses. Another measure that was tested to improve the controllability of the reactive distillation process [15,53] and to avoid the excessive foam formation was the thermal insulation of the whole system until the Liebig condenser, using double bubble reflective aluminum foil. Even the use of hot water was tested in the Liebig condenser to have a smoother drop of pressure throughout the distillation setup (Figure 2) and to promote the formation of the NH_4_HCO_3_ crystals in the RB flask at room temperature, where the distillate was collected.

### 2.3. Design of Experiments

The initial design of the experiments was carried out to investigate the effect of the boil-up rate on the isolation efficiency of NH_4_HCO_3_ from the anaerobic digestate with the batch distillation apparatus [54]. Emphasis was placed on the analysis of the transient states of heating up and cooling down, as these would offer the best conditions to promote the volatilization of CO_2_ and NH_3_ while minimizing the vaporization of H_2_O [8] and the production of diluted condensates [54]. This also implied a thorough assessment of the suitability of the batch distillation setup for the processing of different types of anaerobic digestates. The mass balances for the different modifications of the apparatus were calculated based on how much weight reduction of the anaerobic digestate was observed as distillate, condensate, or NH_4_HCO_3_ crystals recovered. Limitations of the isolation of NH_4_HCO_3_ by this method were highlighted. The composition of the crystals of inorganic fertilizer was confirmed with the Cary 630 Fourier-Transform Infrared (FTIR) Spectrometer of Agilent Technologies, with Attenuated Total Reflectance (ATR) sampling module. The change in the composition of the anaerobic digestate for being subjected to the distillation process was characterized with the titration methodology reported elsewhere [61,62]. Since the ammonium bicarbonate is the main substance responsible for the buffer capacity of the anaerobic digestate, the extraction of this compound implies a transfer of the buffer capacity from the NH_4_HCO_3_-depleted digestate to the NH_4_HCO_3_-concentrated distillate. The results obtained with all these experiments were suitable for the design of continuous processes. For example, a flash distillation could be designed by considering that the equilibrium that was reached in the batch setup after a certain time of operation could be attained in the flash tank instantaneously while processing a flow rate of 80 mL digestate divided by the time of the batch operation.

#### 2.3.1. Processing of AWD

The traditional batch distillation apparatus [54] was operated in different modes, depending on the type of anaerobic digestate or model solution. Particularly, the processing of AWD was optimized based on the dose of antifoam, temperature, and duration of the distillation. For the AWD, operations of 10, 20, and 30 min at temperature over 90 °C were tested with antifoam (~850 ppm [54]) due to the high heating rate (>5 °C/min) during the ramp-up period, and because the direct isolation of the NH_4_HCO_3_ crystals was not possible. The only route for depleting the AWD was the production of a highly concentrated condensate that still required cooling at 3 °C and addition of acetone for precipitation of NH_4_HCO_3_ crystals [61]. Additionally, conditions available in the AD plants were considered to maximize the synergies and minimize the cost of the downstream processing of the anaerobic digestate [54]. In this way, the development of a novel circulating NH_4_HCO_3_ harvesting system was attempted, and this would be applied to the floating cover of the pasteurization tanks at 70 °C of the AD plants. At lab scale, this system for conveniently harvesting the NH_4_HCO_3_ crystals in the batch distillation setup [54] was investigated [62,63].

#### 2.3.2. Processing of FWD

The transient conditions were more suitable for isolating the NH_4_HCO_3_ from the FWD directly, without using antifoam [64]. Low heating rates (<5 °C/min during the ramp-up period) were tested for direct isolation of NH_4_HCO_3_ crystals of the FWD, by means of operations that could last up to 8 h without antifoam. The distillation temperatures ranged between 95 and 99 °C, but the direct formation of the NH_4_HCO_3_ crystals while processing the FWD was mainly observed during ramp-up. While temperature of the volatilizing gases was <95 °C, the emissions of CO_2_ and NH_3_ were prioritized over H_2_O volatilization [8]. Additional combinations tested included the direct NH_4_HCO_3_ recovery and subsequent addition of antifoam to the half-depleted FWD to produce a concentrated distillate. Furthermore, different times of operation and allowing the NH_4_HCO_3_ crystals, which were generated in a previous run, to serve as seeds for enhancing the solid phase formation in the subsequent batch distillations were investigated.

#### 2.3.3. Optimization of the Isolation of NH_4_HCO_3_ by Adding Titrants

The validation of the results previously obtained with the model of Aspen Plus^®^ v12 (Aspen Technology, Inc., Bedford, MA, USA) [8], with regard to improving the reactive distillation process [15,53] by adding acid or basic titrants (0.06 M HCl or 0.13 M NaOH), was tested with a model solution of approximately 10 g NH_4_HCO_3_/L, which was equivalent to the AWD, but without the inconvenience of excessive foam formation. Elucidating the effect of a pH conditioner in producing a concentrated condensate of NH_4_HCO_3_ was anticipated to be challenging since CO_2_ volatilization was promoted at low pH and NH_3_ volatilization was promoted at high pH.

#### 2.3.4. Descriptive Statistical Analysis

Based on the understanding that different approaches of conducting the distillation should be carried out for FWD and AWD (Table 1), a single replicate was carried out for each experimental condition, with the aim of evaluating as many conditions as possible by tuning parameters. In case more than a single replicate was carried out, the average values were plotted along with the standard deviation as error bands. The purity of the product isolated was determined by acid–base titration of the distillates (concentrated NH_4_HCO_3_ aqueous solutions) which resolved the 3 alkalinity peaks (OH, P, and M). In case the solid crystals of NH_4_HCO_3_ were isolated, the comparison of their composition to the reagent-grade commercial chemical was made by FTIR analysis. The differences in the titration curves and FTIR spectra that characterize the performance of the isolation of NH_4_HCO_3_ out of the anaerobic digestates were identified by descriptive statistics and by visual inspection of the plots.

## 3. Results

### 3.1. Characterization of the Batch Distillation Equipment

The batch distillation setup [54] did not have the optimum design for processing anaerobic digestates (whole or liquors), although it was easy to disassemble and clean. This apparatus did not provide comprehensive control over the variables that were intended to be manipulated (i.e., heating power, reflux, and doses of acid and base titrants) to affect the efficiency of the separation and isolation of NH_4_HCO_3_ of the AWD and FWD. The inertia of the system was given by the fact that the temperature was not measured directly in the anaerobic digestate, and the bulb of the thermometer was in touch with the glassware that kept the headspace of the distillation apparatus closed. To ensure better contact between the glassware of the batch distillation setup and the bulb of the thermometer, several drops of mineral oil were introduced into the pit that held the thermometer. Despite this enhancement in the conduction of the heat, the temperature displayed by the thermometer still depended on the rate of the volatilizing gases (convective moment of gases) and the heat losses to the surrounding environment of the fume hood. The foam formation was found to be a mechanism of the complex aqueous suspension, as it was the anaerobic digestate, to prevent the volatilization of gases. As more foam was observed, the temperature marked in the thermometer started to decrease because the rate of the volatilizing gases was lower. Both the inertia of the system and the physicochemical mechanism of the foam formation were the two main constraints that hindered the downstream processing of the anaerobic digestate from obtaining a more stable soil organic amendment by removing CO_2_ and NH_3_, and efficiently isolating the NH_4_HCO_3_ as a commercial-grade inorganic fertilizer [50,51]. The system was insulated with reflective bubble aluminum foam, as lining with this material would minimize heat losses and inertia, and the occurrence of maximized equilibria in the headspace [54]. A lower dose of Dispelair^®^ K989 4308 L was required to avoid the excessive foam formation, compared to the requirements of Dispelair^®^ DP 681 3281 L for the same purpose [54]. However, operating this complex system was still difficult and it was necessary to stop the experiment several times. The distillation could be stable for a while, with the continuous dropping of distillate, and later became uncontrolled [55], in line with the physico-chemical mechanism that prevented the progressive depletion of the anaerobic digestate by extracting CO_2_ and NH_3_.

### 3.2. Processing of AWD

**Figure 3:**
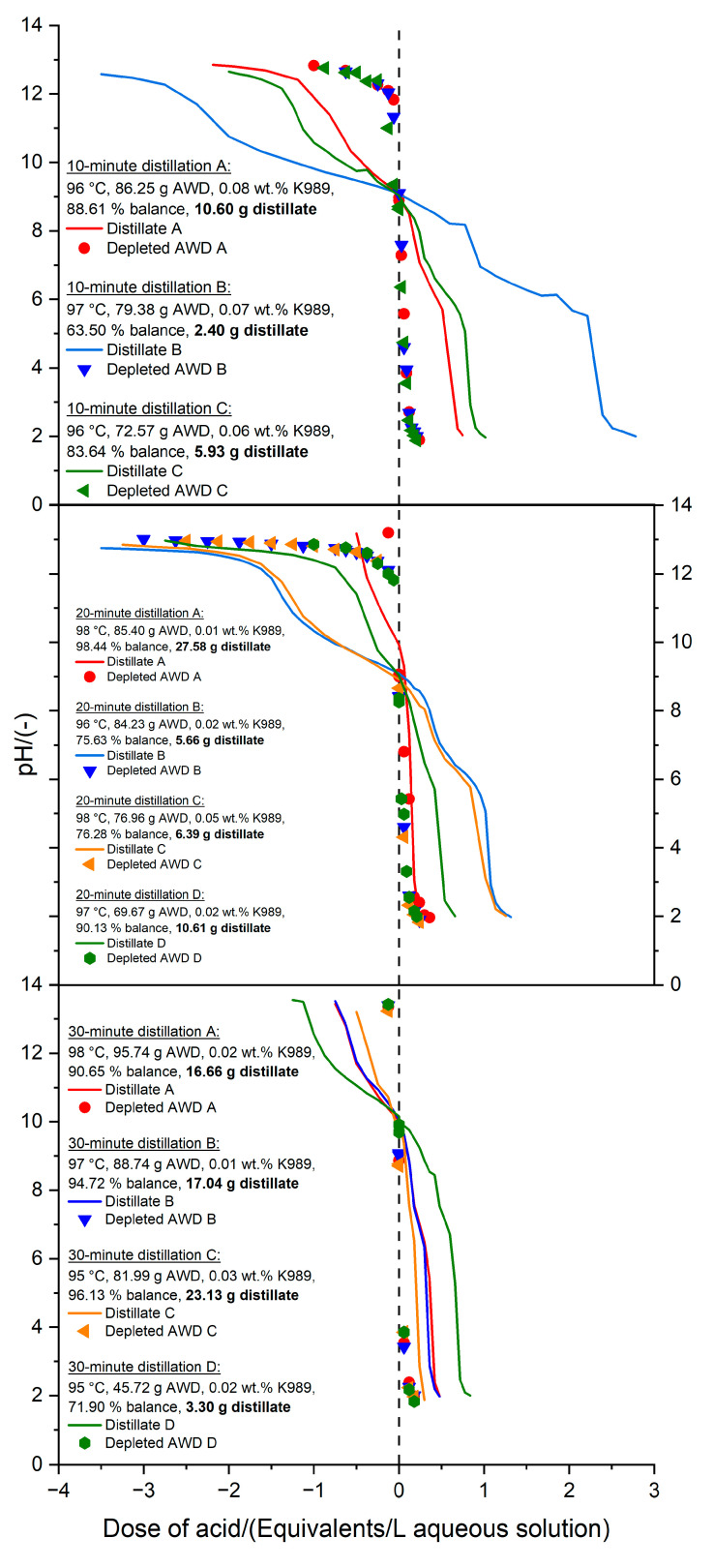
Titration curves of the distillates and the depleted AWD after distillations of 10 min, 20 min, and 30 min. The numbers of replicates were 3 for the 10 min distillation, 4 for the 20 min distillation, and 4 for the 30 min distillation. Due to the complexity of the system, different amounts of distillate were produced, and this is highlighted in bold letters in the legends.

Figure 3 shows the results for the different times (10, 20, and 30 min) devoted to the batch distillations of AWD. The most accurate representation of the extent of the distillation process was given by the titration of the distillate and the spent digestate. CO_2_ might have volatilized earlier than NH_3_, since only the P-alkalinity (at pH 9) can be seen in the spent digestate after a 30 min distillation [61]. The phenolphthalein (P) was the only type of alkalinity (at pH 9) associated with the presence of NH_4_^+^-N, while the OH-alkalinity (at pH 12) and the M-alkalinity (at pH 6) completely disappeared by the end of the 30 min distillation (Figure 3). Furthermore, comparing the different trials for each length of distillation, Figure 3 shows that the fewer amounts of distillate produced, the greater the buffer capacity because the aqueous solution was more concentrated in NH_4_HCO_3_. With these data, it was possible to think about a continuous system, where the key parameters were the heating rate and the residence time. At 30 min of distillation, more CO_2_ and NH_3_ were lost because although the buffer capacity of the depleted digestate decreased, the buffer capacity of the distillate did not increase proportionally. The traditional distillation setup (Figure 2) [54] had a small hole after the condenser to allow the operation at atmospheric pressure, and this could have also led to losses of NH_3_ and CO_2_ during distillations (>3 h).

**Figure 4:**
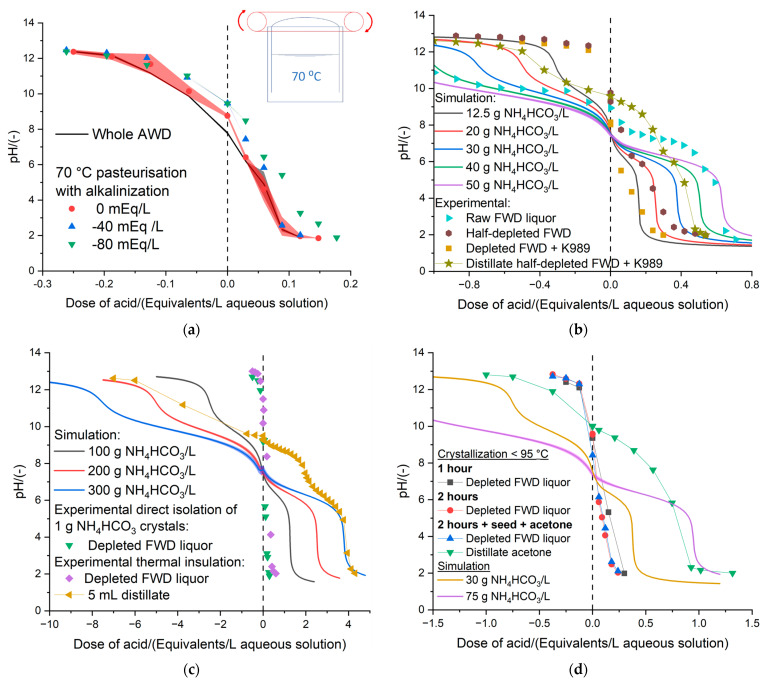
(**a**) Titrations of the depleted AWD after removing NH_4_HCO_3_ in the pasteurization process [63,64] using different levels of alkalinization (negative values of milliequivalents of acid applied per liter of AWD) to further enhance the sanitization of the digestate. (**b**) Serial distillation of ~80 g raw FWD liquor for isolating 3 g NH_4_HCO_3_ crystals before adding the antifoam Dispelair^®^ K989 to completely deplete the FWD and recover ~20 g distillate with approximately 30 g NH_4_HCO_3_/L. The plot includes simulations in Aspen Plus^®^ v12 [66] to characterize the concentration of NH_4_HCO_3_ in aqueous solutions. (**c**) Comparison of the processing strategies of operating at low heating power to enable the direct crystallization of 1 g NH_4_HCO_3_ in the distillation apparatus with the glass walls at room temperature [64] with the production of 5 mL of highly concentrated distillate in a thermally insulated setup. This plot includes simulations in Aspen Plus^®^ v12 [66] to characterize the concentration of NH_4_HCO_3_ in aqueous solutions. (**d**) Comparison of different processing strategies for isolating the NH_4_HCO_3_ of the FWD liquor by distillations at temperature below 95 °C, lasting for 1 h and 2 h. The 2 h distillation was also tested with acetone and NH_4_HCO_3_ crystals already in the condensation area of the distillation setup, to act as seeds and to enhance the fixation of NH_3_ and CO_2_.

In order to minimize the need for cooling and adding antisolvent, the direct synthesis of the NH_4_HCO_3_ crystals was investigated with the batch distillation apparatus [54] and using the FWD [63]. AWD had less NH_4_^+^-N (Figure 1), thus it required greater heating to enable the separation of NH_4_HCO_3_-H_2_O and required antifoam [54]. In the case of the FWD, it was feasible to isolate the crystals of NH_4_HCO_3_ directly by operating at low heating rates, without the addition of antifoam (e.g., silicone oil) [54,64]. Reducing the heating load reduced the separation rate and avoided the occurrence of the physico-chemical mechanism of foam formation, which would otherwise prevent the volatilization of gases. The alkalinization [65] was considered to collect the data of Figure 4a, which also includes a schematic of a novel circular system for harvesting the NH_4_HCO_3_ crystals in the pasteurization tank [61]. The direct isolation of the NH_4_HCO_3_ crystals was previously attempted with the whole AWD, emulating the conditions of pasteurization, but without using the thermometer in the headspace to harvest the crystals of NH_4_HCO_3_ [64]. The greatest crystallization of NH_4_HCO_3_ in the thermometer was found when the AWD and the FWD were kept for a longer time before starting to swap the thermometers [65]. Therefore, the most important parameter for the extraction of NH_4_HCO_3_ from the AWD and the FWD was the residence time in the pasteurizer. The addition of an alkali to the AWD negatively affected the crystallization in the thermometer. Figure 4a shows that more acid was required to reach the titration endpoint (pH < 2), but this should not be associated with a lower depletion of the AWD during the pasteurization process coupled with NH_4_HCO_3_ removal. When the operation of AWD was compared to the processing of FWD [63], mass balances were 34.16 ± 10.88% and 48.09 ± 12.90% for the pasteurization of AWD and FWD, respectively. The amounts of condensates produced were 0.57 ± 0.24 g and 0.61 ± 0.24 g for the 166.00 ± 21.21 g AWD and 165.00 ± 36.77 g FWD, respectively, in an hour pasteurization [54].

### 3.3. Processing of FWD

Figure 4b shows the results of operating the distillation at a low heating rate to enable the direct isolation of NH_4_HCO_3_ crystals and subsequently adding Dispelair^®^ K989 antifoam to the half-depleted FWD to operate at a higher heating power and produce a distillate. Figure 4b shows that the raw FWD liquor had a greater buffer capacity (i.e., a more horizontal titration curve) than the half-depleted FWD, but the latter had a greater buffer capacity than the depleted FWD. This was the expected trend, as less NH_4_HCO_3_ remained in the FWD as the distillation progressed. The raw FWD liquor contained approximately 50 g NH_4_HCO_3_/L, as displayed in Figure 4b by comparing the experimental titration curve with the simulation in Aspen Plus^®^ v12 [66]. Since approximately 80 g of raw FWD liquor was introduced in the 250 mL RB flask at the beginning of the distillation and ~3 g NH_4_HCO_3_ was isolated in the first part of the distillation, it was anticipated that the remaining half-depleted FWD had a concentration of approximately 12.5 g NH_4_HCO_3_/L. Figure 4b confirms that, on average, this was approximately the content of NH_4_HCO_3_ because although the M-alkalinity (at pH 6) was slightly higher in the half-depleted FWD, the P and OH alkalinities (at pH 10 and 12, respectively) were lower than what was expected theoretically. In general, there was a significant difference between the experimental titration of the samples and the characterization of the model aqueous solutions of NH_4_HCO_3_ with Aspen Plus^®^ v12, as could be seen at the point of the zero-dose of acid that shows pHs of 9.03 ± 0.66 and 7.55 ± 0.07 for the experimental and simulative/theoretical results, respectively (Figure 4b). Approximately 20 g of distillate was produced after adding the K989 antifoam to the half-depleted FWD, and this condensate had approximately 30 g NH_4_HCO_3_/L (Figure 4b), which implies that ~0.6 g NH_4_HCO_3_ was transferred from the half-depleted FWD to the distillate and the depleted FWD remained with a content of ~5 g NH_4_HCO_3_/L (Figure 4b).

Figure 4c compares the processing strategies of directly isolating 1 g NH_4_HCO_3_ from ~80 g FWD liquor [64], meeting the mass balance in 37.23%, to the production of ~5 mL saturated distillate, and attaining a 66.76% mass balance. Mass balances over 95% could be attained by allowing the sufficient formation of the distillate, since more NH_3_ and CO_2_ would be absorbed in the aqueous solution. The losses of NH_3_ and CO_2_ were not entirely responsible for the lower mass balance when operating at a lower heating rate, but also the crystallization of NH_4_HCO_3_ through the distillation setup (Figure 2) [54], particularly in locations from which this inorganic fertilizer was difficult to retrieve and quantify [64]. The formation of dew or NH_4_HCO_3_ crystals in the glass surface of the distillation apparatus was investigated as the most convenient technique for isolating the inorganic fertilizer because the needs for the subsequent cooling of the distillate and addition of antisolvent would be avoided. A compromise could be attained by operating at a low heating power [64], which allowed the crystallization of NH_4_HCO_3_ in the distillation apparatus, and subsequently enhanced the volatilization of H_2_O to clean the walls of the setup and create an aqueous solution with much more alkalinity than if H_2_O was volatilized from the beginning to create a diluted distillate. Figure 4c shows that the saturated distillate had the alkalinities of a saturated aqueous solution of NH_4_HCO_3_ [8]. It should be noted that an enhancement in the heating power while keeping the distillation under control (i.e., minimum foam formation) could be only achieved by applying thermal insulation on the distillation apparatus. Additionally, as the thermal insulation minimized the heat losses to the surrounding environment of the fume hood, it also favored the dissolution of any crystals in the distillation apparatus, and the NH_4_HCO_3_ could be conveniently recovered in the saturated distillate. The 5 mL distillate (Figure 4c) with a very high buffer capacity was obtained by promoting the volatilization of H_2_O and washing away the NH_4_HCO_3_ crystals stuck to the walls of the glass distillation setup (Figure 2).

Figure 4d elaborates on the approach of the direct isolation of NH_4_HCO_3_ crystals by testing different times of operation and allowing the crystals, which were generated in the previous run, to serve as seeds for enhancing the solid phase formation in the subsequent batch distillations. Additionally, 12 mL of acetone was added to the RB flask, that remained at room temperature, where the distillate was supposed to be collected, to enhance the formation of NH_4_HCO_3_ crystals in this part of the distillation apparatus. This supersaturated acetone distillate soon turned into a reddish color and presented a concentration between 30 and 75 g NH_4_HCO_3_/L (Figure 4d). The mass balance matches were 39.15%, 10.45%, and 48.64% for the distillation of 1 h, 2 h, and 2 h with NH_4_HCO_3_-seed and acetone. The operation of 1 h had fewer losses than the operation at 2 h, but the fixation of NH_4_HCO_3_ could be improved with the presence of seeds and by adding acetone. In general, the mass balances were poorly achieved because there were losses and the crystals did not only end up in the RB flask, but in the walls of the whole setup (Figure 2), which was very difficult to recover and quantify. Also, there was not enough aqueous liquid phase for all gases to be trapped. The best settings for the SLS Lab Basics 280c Hotplate Stirrer to promote crystallization directly were 130 °C and 100 rpm. These settings were found to give a low heating rate to prevent excessive foam formation, but still represented sufficient power (heating up rate of 2 °C/min at 60 °C) to enable the NH_4_HCO_3_ separation from the FWD liquor via direct crystallization [64] in the batch distillation apparatus (Figure 2) [54].

### 3.4. Optimization of the Isolation of NH_4_HCO_3_ by Adding Titrants

**Figure 5:**
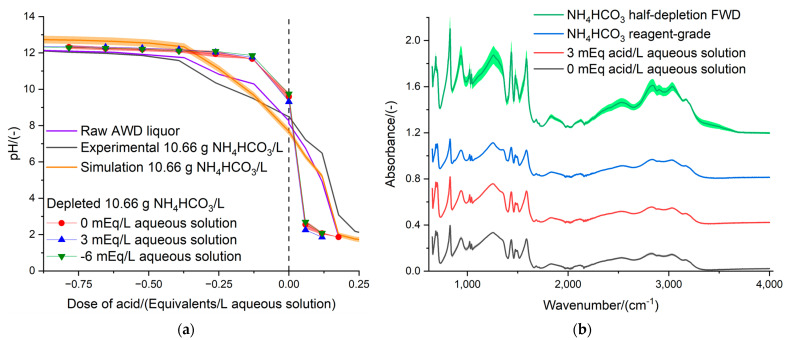
(**a**) Optimization of the isolation of NH_4_HCO_3_ from model solutions of 10.66 g/L, which behaves similarly to the AWD, by adding 3 mEq acid or 6 mEq base per liter of feedstock, for comparison purposes with the theoretical results of the previous investigation [8]. (**b**) Comparison of the FTIR spectrum of the commercial-grade NH_4_HCO_3_ to the crystals isolated from different distillation processes: half-depletion of FWD (Figure 4b) and optimization of the distillation by adding 3 mEq/L dose of acid (Figure 5a).

Figure 5a shows the results of the distillations of 10.66 g NH_4_HCO_3_/L aqueous solutions with different levels of acidification and alkalinization to validate the results of the previous investigation [8], where the isolation of NH_4_HCO_3_ from the anaerobic digestate was optimized with Aspen Plus^®^ v12 simulations. Figure 5a also includes the titration curve of the raw AWD to show that this organic amendment has the same buffer capacity as the 10.66 g NH_4_HCO_3_/L model aqueous solution. The model aqueous solution of 10.66 g NH_4_HCO_3_/L was the most suitable option for optimizing the production of NH_4_HCO_3_ by carefully tuning the parameters, such as the dose of acidifying and basifying agents. Otherwise, using the AWD would produce many physico-chemical reactions (e.g., foam formation) that prevent the experiments from running smoothly. However, it was not possible to significantly differentiate the spent digestate that resulted from the normal distillation of 10.66 g NH_4_HCO_3_/L (0 mEq/L) to those obtained from the distillation using 3 mEq acid/L and 6 mEq base/L as conditioners of the feedstock. This implies that the three distillations removed similar amounts of NH_4_HCO_3_ from the model solutions; thus, the addition of a pH conditioner did not improve the extraction process. The processing of real samples of anaerobic digestates was not suitable for the validation of the theoretical results from a previous investigation on integrating the recovery of NH_4_HCO_3_ via flash distillation in a continuous AD process [8] (Figure 5a). Nevertheless, it was possible to confirm that the NH_4_HCO_3_ crystals isolated from the model solutions of 10.66 g NH_4_HCO_3_/L and the FWD had similar compositions (Figure 5b), and these were comparable to the commercial-grade NH_4_HCO_3_ [50,51]. The lower stability and greater availability of the valorized inorganic fertilizer to crops was proven with the FTIR analysis. The NH_4_HCO_3,_ which was isolated directly from the FWD (green line of Figure 5b), had an enhanced FTIR spectrum. The difference in the height of the peaks should be related to more stretching and bending vibrations at a higher energy level due to the greater instability and availability of NH_4_^+^-N for crops, rather than to a more concentrated sample [61]. It is noteworthy to mention that the NH_4_HCO_3_ crystals of the FWD released an intense smell, like H_2_S, and was more readily available to plants, degradable, and unstable than the same inorganic fertilizer from a different source (e.g., commercial NH_4_HCO_3_ from the supplier ThermoFisher Scientific Ltd.). Thereby, the thermal distillation treatment of the anaerobic digestate could be regarded as a mitigation technology that prevents pollution swapping, depending on whether the rate of release of the NH_4_^+^-N, upon the land application of the inorganic fertilizer, would be sufficiently slow to be absorbed by plants through their roots while preventing gaseous emissions.

## 4. Discussion

A compromise between the concentration and the volume of condensate generated was the key dependent variable for optimizing the removal of NH_4_HCO_3_ from the anaerobic digestates via reactive distillation [15,53]. A highly concentrated distillate minimized the need for downstream processing (i.e., less requirement for cooling, antisolvent, or operating above the atmospheric pressure [16,17] to induce supersaturation and precipitation). Acetone was found to perform better than isopropanol when inducing the supersaturation of the NH_4_HCO_3_ aqueous solutions. After adding both organic solvents, small bubbles were observed due to the supersaturation, but in the case of using isopropanol as the antisolvent, the gas release was greater, and fewer crystals of NH_4_HCO_3_ precipitated. Although the isopropanol is cheaper than acetone (i.e., approximately GBP 1.5/L cheaper), the lower cost of the antisolvent cannot be justified by the lower recovery of the NH_4_HCO_3_ crystals. Ukwuani and Tao [47] used ethanol to induce the precipitation of (NH_4_)_2_SO_4_, and this could also be suitable to induce the precipitation of NH_4_HCO_3_. From a health and safety perspective, the toxicities of acetone, isopropanol, or ethanol are much lower than other organic solvents, such as dichloromethane or tetrahydrofuran. Minimizing the use of antisolvent by producing highly concentrated condensates might also imply losses of NH_3_ and CO_2_ because of the lower volume of the aqueous phase available for the absorption of these gases. In practice, the direct isolation of the NH_4_HCO_3_ crystals is very dependent on the composition of the anaerobic digestate because the organic soil amendment should have a very high content of inorganic N and C (Figure 1) for this purpose. Additionally, transient conditions of heating up while the temperature remains below 90 °C (to minimize the evaporation of water while maximizing the volatilization of CO_2_ and NH_3_) were found to be required conditions. A more advanced apparatus needs to be developed to have closer control of all the independent variables, including the heating up rate, the condensation temperature, and the location of the condensation. It is important to clarify that, according to the Royal Society of Chemistry [67], the bulb of the thermometer should be in the path of the vapor [54] and if this is too low or too high, an inaccurate value for the boiling point would be determined.

The direct formation of NH_4_HCO_3_ crystals could only be significantly assessed and quantified with the FWD. According to the review of Yang et al. [19] and our own previous investigation [61], anaerobic digestates obtained from bioreactors fed with animal manure could have an even greater content of NH_4_^+^-N than FWD; hence, they could be also suitable for directly obtaining the NH_4_HCO_3_ crystals using a simple setup [54,62]. The distillation process was more suitable for the AWD, but it was more expensive because it required more heating of the anaerobic digestate, cooling of the condensate, and an antisolvent to enable crystallization. Additionally, the specifications of the distillation process hardly match the operating conditions of an AD plant [61] and could not be synergistically implemented as part of the existing equipment. A previous investigation described how the buffer capacity of the digestates and the biogas-pipeline condensates were similar because both solutions were in equilibrium, especially under the conditions of the pasteurizer [61]. It is noteworthy to mention that the energy consumption for the pasteurization or distillation could be cancelled by considering the cost of inorganic acids or alkaline agents, which otherwise would be required for the sanitization process [68]. Performing the distillation with the lab setup (Figure 2) [54] allowed us to obtain a greater buffer capacity in the condensate than in the spent/depleted distillate (e.g., Figure 3 and Figure 4b). This was the reason for which characterizing only the spent digestate after the distillation was found more convenient, since it was a quicker evaluation of the performance of the distillation (e.g., Figure 4a and Figure 5a), assuming that there were no losses, and all the gases were fixed as NH_4_HCO_3_ crystals or absorbed in the distillate. Residue curve maps are profiles of compositions in the bottom flasks during the transient operations and zero reflux, which are often developed to study the feasibility of a particular separation. The data herein (i.e., titration curves) could be end parts of the residue curve maps [15,69], although these titration curves of the depleted digestate during batch distillation only describe the composition at the end of the batch operation.

The Liebig condenser was also operated with hot water (70 °C) to allow the NH_4_HCO_3_ crystals’ formation at a convenient location for harvesting: the RB flask at room temperature at the edge of the batch distillation apparatus. The fact that it was possible to directly isolate the crystals of NH_4_HCO_3_, without the production of a liquid distillate, could imply that the reaction of NH_3_, CO_2_, and H_2_O took place in the gaseous phase, following a deposition process to form the solid crystals of NH_4_HCO_3_ [70]. In fact, Lee et al. [70] reported the deposition of the NH_4_HCO_3_ crystals along with sulfur compounds, which agreed with the strong smell observed in the NH_4_HCO_3_ crystals isolated from the FWD (Figure 5b). The reddish color of the aqueous solution of NH_4_HCO_3_ mixed with acetone for 4 h could be anticipated, based on the information provided by Govindan et al. [71]. In the same way, Overcashier et al. [72] also confirmed the rapid decomposition and sublimation of NH_4_HCO_3_ [73]. It would be necessary to investigate solid surfaces or (hollow) membranes [74] made of polypropylene, polyvinylidene fluoride, or polytetrafluoroethylene [75], to come up with a novel design for solids that could promote the deposition of NH_4_HCO_3_ for a better recovery of this compound, avoiding the requirements of antisolvent-induced precipitation in the liquid aqueous distillate. Operational challenges still need to be overcome because even at a smooth heating rate of 3 °C/min, foam appeared and the temperature of the thermometer in the batch distillation dropped due to the lower rate of volatilization of compounds from the anaerobic digestate.

Given the negative effect of the magnetic stirring, it might be more suitable to enhance the distillation with the use of antibumping agents, that provide nucleation sites and lead to a smooth and uniform boiling of the digestate (whole or liquid) [67]. Another measurement to control the excessive rise of foam would be the addition of baffles in the headspace [76,77]. The chemistry of proprietary blends of polymeric esters of ethylene oxide and/or propylene oxide is widely used in the wastewater treatment industry. The Dispelair^®^ DP 681 product is designed to control large industrial-scale digesters (>100 m^3^), where low dosages are often achievable with a continual background addition by a pump (i.e., the 1–10 ppm is only a guideline). The simple silicone emulsion gave a foam knockdown effect, and it was a more effective antifoam agent since a lower dose of Dispelair^®^ K989 was necessary than that of Dispelair^®^ DP 681. This was in agreement with the recommendation of using silicon oil for the distillation process of Drapanauskaite et al. [16].

An attempt to use a solid organic absorbent material to recover the inorganic N and C from the distillate and to granulate and coat the NH_4_HCO_3_ crystals offered promising results [78]. The depleted anaerobic digestate liquor that remained after the distillation was dehydrated and was used as a binding agent to produce the granules and to improve the stability of the NH_4_HCO_3_ and avoid pollution swapping [52,79]. The coating or granulation of the NH_4_HCO_3_ crystals with the organic solid absorbent is necessary to improve the stability of the inorganic fertilizer [52,78]. Furthermore, since the hydration of the binding agent is required for molding, maturation, curation, and self-hardening, the direct combination of this material with the distillate or condensate is possible, assimilating the dissolved NH_4_HCO_3_ in the complex organomineral matrix, and avoiding the cooling and antisolvent-induced precipitation steps. The results of the compressive strength testing of these 2 cm diameter pellets of concentrated liquor and crystals of ammonium bicarbonate were (*n* = 8) 198.57 ± 58.43 N and 0.94 ± 0.75 mm [79], which confirmed the superior mechanical properties of the concentrated liquor as the binding agent because the minimum compressive strength for granular fertilizer should be around 50 N [80,81]. The pellets of the dehydrated liquor offered a very high buffer capacity due to the high concentration of alkali and alkaline earth minerals, which in the raw liquor, with 98% moisture, would not have the same effect. The assessment of the nutrient enrichment of the granules with the NH_4_HCO_3_ would require prior water-soluble extraction for 1 h and 100 rpm, to have a more homogenous liquid phase that could be titrated easily. This extraction with water (or another matrix, such as an aqueous solution of KCl or CaCl_2_ [82,83,84]) gives a valid representation of the nutrients available to plants once the organo-mineral fertilizer is applied to land [85].

This way of handling the NH_4_HCO_3_ could minimize its effect as a fumigant, which is widely reported in the literature [86,87,88,89,90,91,92]. The NH_4_HCO_3_ isolated out of the FWD had greater ammonia availability than the commercial product, so it is expected that this recycled material has a greater effect as a fumigant. The fumigant role should not be understood as toxicity-related because the ammonium bicarbonate can act as a nutrient source for plants, once the NH_4_^+^-N is converted to a nitrate form. Plants withering is not always related to fungal infections or soil-borne plant pathogens, but due to hostile conditions such as a lack of moisture or nutrients. This all makes the NH_4_HCO_3_ a very valuable material to ensure the crop growth by the dual action of fertilization and fumigation.

The validation of the theoretical results of the previous investigation [8] using acid doses of 0.6 Eq/L and −0.9 Eq/L was not confirmed, and no ameliorations in isolating the NH_4_HCO_3_ and depleting the anaerobic digestates were found (Figure 5a). The acid doses tested experimentally were much lower (i.e., 3 and −6 mEq/L; Figure 5a) than those found as the optimum in the integrated model in Aspen Plus^®^ v12, considering an anaerobic digestate with 50% moisture, because the 10.66 g NH_4_HCO_3_/L model solution (i.e., with a buffer capacity equivalent to the AWD; Figure 5a) had a water content of 98.93%. Expressed on a dry matter basis, the optimum acid doses found with Aspen Plus^®^ v12 for the isolation of NH_4_HCO_3_ via distillation were 1.18 and −1.82 Eq/kg [8], while the acid doses tested in the present investigation were 0.28 and −0.56 Eq/kg. The optimization of the processing of the anaerobic digestate, by adding acidic or basic agents, should be investigated once the controllability of the setup has improved enough to closely monitoring the temperature of distillation and to handle the inconveniences of foam formation.

## 5. Conclusions

Traditional distillation was suitable for improving the management of NH_4_^+^-N of the anaerobic digestate, producing a commercial-grade fertilizer and stabilizing this organic manure for subsequent storage and land application (i.e., minimizing losses of NH_3_ via volatilization). The distillation of FWD with a greater content of NH_4_^+^-N allowed the direct isolation of the NH_4_HCO_3_ crystals, while the processing of AWD required a dose of antifoam of ~850 ppm to isolate an aqueous solution of NH_4_HCO_3_. The reason was that the extraction of the NH_4_HCO_3_ from the AWD required a higher temperature (>90 °C) and duration (i.e., steady batch operation) than the recovery of the inorganic fertilizer from the FWD. Moreover, the problem of obtaining the aqueous solution of NH_4_HCO_3_ was that cooling at 3 °C and the addition of acetone as an antisolvent was necessary to induce the supersaturation and precipitation of the solid crystals. The isolation of NH_4_HCO_3_ from the FWD was attained in a transient mode at temperature below 90 °C (i.e., while heating up to reach the desired distillation temperature or cooling down once finished with the batch distillation). For the operating conditions to be regarded as techno-economically feasible, they should be attained in the AD plant by harvesting the heat from the engines, which convert the biogas into electricity. Based on the obtained results, the stabilization of FWD by NH_4_HCO_3_ extraction could be attained with minor modifications of the pasteurization tank at 70 °C (i.e., including a circulating harvesting system) present in AD plants. The system to harvest NH_4_HCO_3_ conveniently from the distillation apparatus and solid surfaces, which foster crystallization, needs more development. The NH_4_HCO_3_ obtained directly from the distillation setup, in the case of FWD, or precipitated from the aqueous solution distillate, in the case of the AWD, presented poor stability, and granulation and coating need to be further investigated to improve the role of this material as a slow-release fertilizer and control its fumigating power.

## Figures and Tables

**Table 1:** Variation range of each parameter investigated for each sample.

Parameter	FWD	AWD	NH_4_HCO_3_ Model Solution *
Temperature/(°C)	>70	95	95
Time/(h)	3	>1	1
Antifoam/(ppm)	-	>850	-
Acid dose/(mEq/L)	-	-	−6–3
Distillate form	Pure NH_4_HCO_3_ crystals	Concentrated NH_4_HCO_3_ aqueous solution	Concentrated NH_4_HCO_3_aqueous solution
Distillate characterization	FTIR	Acid–base titration	Acid–base titration

* Used to model the distillation of anaerobic digestate without the interference of other factors that affect the performance (e.g., foam formation).

## Data Availability

Research data are publicly available as videos on YouTube [54,59,61,63,66,72,77] and as Aspen Plus^®^ models hosted by ZENODO [65].

## Abbreviations

AD	anaerobic digestion
ATR	attenuated total reflectance
AWD	agrowaste digestate
C	carbon
CAPEX	capital expenditure
CHP	combined heat and power
CO_2_	carbon dioxide
FTIR	Fourier-transform infrared
FWD	food waste digestate
H_2_SO_4_	sulfuric acid
mol%	mol percentage
N	nitrogen
NH_4_^+^-N	ammoniacal nitrogen
(NH_4_)_2_CO_3_	ammonium carbonate
NH_2_COONH_4_	ammonium carbamate
NH_4_HCO_3_	ammonium bicarbonate
OPEX	operational expenditure
RB	round-bottom
vol.%	volume percentage
